# Motion-Based Object Location on a Smart Image Sensor Using On-Pixel Memory

**DOI:** 10.3390/s22176538

**Published:** 2022-08-30

**Authors:** Wladimir Valenzuela, Antonio Saavedra, Payman Zarkesh-Ha, Miguel Figueroa

**Affiliations:** 1 Department of Electrical Engineering, Faculty of Engineering, Universidad de Concepción, Concepción 4070386, Chile; 2 Embedded Systems Architecture Group, Institute of Computer Engineering and Microelectronics, Electrical Engineering and Computer Science Faculty, Technische Universität Berlin, 10623 Berlin, Germany; 3 Department of Electrical and Computer Engineering (ECE), School of Engineering, University of New Mexico, Albuquerque, NM 87131-1070, USA

**Keywords:** smart image sensor, smart pixel, vision chip, intelligent sensor, object detection, object location, motion-based, frame difference, field-programmable gate array, very large scale integration

## Abstract

Object location is a crucial computer vision method often used as a previous stage to object classification. Object-location algorithms require high computational and memory resources, which poses a difficult challenge for portable and low-power devices, even when the algorithm is implemented using dedicated digital hardware. Moving part of the computation to the imager may reduce the memory requirements of the digital post-processor and exploit the parallelism available in the algorithm. This paper presents the architecture of a Smart Imaging Sensor (SIS) that performs object location using pixel-level parallelism. The SIS is based on a custom smart pixel, capable of computing frame differences in the analog domain, and a digital coprocessor that performs morphological operations and connected components to determine the bounding boxes of the detected objects. The smart-pixel array implements on-pixel temporal difference computation using analog memories to detect motion between consecutive frames. Our SIS can operate in two modes: (1) as a conventional image sensor and (2) as a smart sensor which delivers a binary image that highlights the pixels in which movement is detected between consecutive frames and the object bounding boxes. In this paper, we present the design of the smart pixel and evaluate its performance using post-parasitic extraction on a 0.35 µm mixed-signal CMOS process. With a pixel-pitch of 32 µm × 32 µm, we achieved a fill factor of 28%. To evaluate the scalability of the design, we ported the layout to a 0.18 µm process, achieving a fill factor of 74%. On an array of 320×240 smart pixels, the circuit operates at a maximum frame rate of 3846 frames per second. The digital coprocessor was implemented and validated on a Xilinx Artix-7 XC7A35T field-programmable gate array that runs at 125 MHz, locates objects in a video frame in 0.614 µs, and has a power consumption of 58 mW.

## 1. Introduction

Computer vision is a discipline that has gained an important place in data analysis on scientific and industrial applications. Among the applications of computer vision are obstacle detection [1] and position and speed estimation for accident avoidance [2] in driverless cars, pedestrian detection using infrared cameras for surveillance [3,4], autonomous underwater monitoring for detecting life on the seabed [5], improvement in the food industry using real-time smart machines and predictable models [6], and real-time pupil localization for driver safety improvements [7,8], among others.

From the point of view of engineering applications, one of the fundamental tasks of computer vision is object detection [9,10]. Object detection has the goal of determining the positions of objects in an image (object location) and determining the semantic categories of each object (classification) [11]. Object detection is not limited to applications in the visible-light range, which can underperform in low-light environments. It is also applicable in the thermal infrared (IR) spectrum, which can increase the robustness of the algorithms under harsh conditions [12,13]. Object detection helps to improve the results of other computer vision methods, and in some cases, it is a necessary step. Some of these methods are face and object recognition [14], recognition of pedestrians in autonomous and assisted driving, object tracking [15], and intruder detection for surveillance and security [16], among others.

Recent works in the literature have shown a growing interest in image and video processing on mobile devices [17], including a variety of approaches for mobile object detection [18,19,20]. One of the key factors of mobile devices in general is portability, mainly supported by a small form factor and a low energy consumption to extend the device’s battery life [21]. Computer vision typically requires a large amount of computation power to be capable of delivering highly precise and fast results and relies on ever-increasing computational requirements for improved accuracy [22,23]. This increment in computational requirements is counterproductive when the goal is to implement computer vision applications on resource-constrained platforms, such as mobile devices or Internet of Things (IoT) endpoints, as the high computational capabilities affect the form factor and power consumption, requiring major efforts to achieve high-performance mobile solutions [24]. Thus, it is important to take into account different considerations when selecting suitable hardware accelerators for computer vision tasks [22].

An important area of interest is the design and use of custom-hardware devices for computer vision to operate in real time [25]. These devices are designed to specifically perform only a few particular computer vision methods [26]. Custom hardware improves energy autonomy, reduces power consumption, and increases hardware integration, which allows small form factors, all this while maintaining competitive performance. These conditions make custom-hardware solutions an attractive approach for mobile computer-vision designs [26].

In the area of custom hardware devices, Smart Image Sensors (SIS) have gained significant attention [27]. SISs are dedicated circuits that combine conventional image sensors, such as CMOS Image Sensors (CIS), with additional processing hardware on the same chip [27], typically at the pixel or column level. When a pixel integrates image acquisition and processing hardware, it is typically referred to as a smart pixel. A smart pixel can perform mathematical operations on the pixel values using digital or analog circuits [28]. Smart pixels enable fine-grained parallelism because they can simultaneously operate on all the image data on the focal plane array (FPA) [27]. The goal of this integration is to have, on a single device, the hardware to capture images and run computer vision algorithms on them, either totally or partially, by executing part of their associated operations. The literature shows several SISs that implement computer vision methods on the visible light spectrum, such as feature extraction [29,30,31,32], edge detection [33,34], face recognition [35,36,37], object detection and tracking [34,38], convolutional neural networks [39], and object detection based on Histogram-of-Oriented-Gradients (HOG) [40]. Other SISs have been designed to operate in the IR spectrum to implement non-uniformity correction and compensation [41,42] and face recognition [43]. Therefore, it is feasible to continue this area of study to expand the available custom-hardware designs.

In this paper, we design and evaluate a novel intelligent Readout-Integrated Circuit (iROIC) and its complete SIS architecture for motion-based object location. The proposed SIS is composed of a smart-pixel architecture based on a Capacitive Transimpedance Amplifier (CTIA) integrator, widely used on thermal IR image sensors. Therefore, the iROIC is suitable for motion-based object location in thermal IR images. As demonstrated in previous work [43], by adding a few extra transistors to the standard CTIA integrator, we can achieve pixel-level image processing that enables low-power, motion-based object location.

We show that a smart pixel can use analog circuits to process video frames during image acquisition. Our results show that motion-based object location can be partially implemented on the pixel, thus avoiding the use of off-chip memory buffers to store the data from the previous frame. As a result, the design computes the frame difference for all the pixels in the imager in parallel during the integration time used to acquire the pixel data. The proposed SIS consists of a heterogeneous architecture composed of an analog stage and a digital stage. On the analog stage, the SIS calculates the difference between consecutive frames, which is the first step of motion-based object location, on the bidimensional smart-pixel array. On the digital stage, the SIS includes a digital coprocessor that computes morphological transformations on the output image and uses a connected-components algorithm to label the objects in the image.

Using a 32 µm × 32 µm pixel, the extra capacitor and switches added to the CTIA to compute frame differences reduce the fill factor (the fraction of the die area dedicated to the photodetector) from 47.6% to 28.1% in a 0.35 µm TSMC process. To evaluate the scalability of our design, we implemented the smart pixel on a 0.18 µm process, achieving a fill factor of 74%, compared to 86.1% for a traditional pixel that uses a conventional CTIA. Using an array of 320×240 pixels, the SIS acquires and computes frame difference at 60 frames per second (fps). Running at 125 MHz, the digital coprocessor uses the frame differences to detect objects in the image in 0.614 µs and consumes 58 mW of power.

The rest of the paper is organized as follows. Section 2 introduces and discusses previous works related to our smart image sensor. We describe the object location algorithm implemented in our SIS in Section 3. In Section 4, we describe the architecture of the smart pixel and the proposed SIS and the architecture of the digital coprocessor. Section 5 describes the resulting area of the smart pixel, the resource utilization of the digital coprocessor, and the simulation results. Finally, Section 6 concludes the paper.

## 2. Related Work

Object-location and classification algorithms are usually implemented on high-performance, high-power hardware platforms such as General-Purpose Processors (CPUs) or Graphics Processors (GPUs) [44,45]. This can be acceptable in a wide variety of solutions but is normally inadequate for low-power applications on embedded or mobile devices. In these cases, special-purpose processing systems on dedicated hardware can achieve high speed and portability with low power. These designs are normally implemented on Field-Programmable Gate Arrays (FPGAs) and Application-Specific Integrated Circuits (ASICs).

In recent years, many FPGA-based Convolutional Neural Network (CNN) architectures have been proposed for object location and classification [46,47,48,49,50]. A common disadvantage of these solutions is the limited capacity of on-chip FPGA memory, which is insufficient to store the large number of parameters required by the CNN. Storing these parameters in external memory reduces the throughput of the implementation, therefore limiting the application of FPGAs for small form-factor object detection in real time. This issue was addressed by Long et al. [51], who implemented an FPGA-based object detection algorithm based on multi-frame information fusion. Their algorithm uses a reduced number of parameters for HOG-based object location and Support Vector Machines (SVMs) for classification, and achieves a throughput of up to 10,000 fps. Nakahara et al. [52] presented an object detection algorithm based on a multiscale sliding-window location search, which binarizes the CNN parameters to reduce their memory requirements, and enables the implementation of the complete network using only on-chip memory. Despite the throughput improvement achieved by using on-chip parameters, all the solutions described above read the image pixels as a serial stream from the image sensor. This has the effect of increasing the latency and limiting the data parallelism available to the algorithm, compared to having access to all the pixels simultaneously. Moreover, algorithms that access the image data serially require line buffers or even entire frame buffers, further increasing the memory requirements of the hardware platform.

As discussed in Section 1, SISs are special-purpose image sensors that combine conventional imagers with additional circuitry to process pixel data on the same chip. When an SIS is designed with computational circuits in every pixel (smart pixels), it can exploit the pixel-level parallelism available in the image-processing algorithm. This shortens latency, increases throughput, and reduces the memory requirements of the solution [30,31,43,53]. To further reduce power and area, SISs typically use analog circuits to store and process the data [30,40]. For example, Lee et al. [40] presented an SIS with embedded object detection that uses a reconfigurable pixel array capable of computing frame differences and spatial gradients. The SIS uses a capacitor in every pixel that acts as an analog memory to compute frame differences. Choi et al [30] use a similar approach to implement motion-triggered object detection. To reduce circuit area and improve fill factor, they use the same capacitor for two horizontally adjacent pixels and alternate its use between odd and even frames, thus trading motion-detection horizontal resolution for fill factor. An alternative technique to improve fill factor is to perform computation during photocurrent integration using an intelligent Readout Circuit (iROIC) [54]. For example, the SIS presented by Gottardi et al. [55] computes local gradients using this technique to implement a lightweight version of local-binary patterns (LBP). Our own previous work [43] also uses an iROIC to compute face recognition in visible-range and IR image sensors using an array of smart pixels based on a configurable CTIA, reducing precision by only 1% compared to a fully digital implementation.

Implementing most of the computation at the pixel level using smart pixels reduces the die area available for the photodector in each pixel, thus reducing the fill factor of the imager. To mitigate this effect, several SISs implement part of the computation at the column level, thus improving fill factor at the cost of reducing parallelism and increasing memory requirements. For example, Jin et al. [33] designed an SIS that computes edge detection using column-level circuits and static memory. Young et al. [56] presented an SIS for object detection that combines pixel- and column-level processing to compute image features based on HOG. Their SIS eliminates redundant illumination data during readout, thus compressing the HOG feature descriptors by up to 25 times compared to a conventional 8-bit readout. Kim et al. [37] detect and recognize faces by combining, on a single chip, a standard imager architecture and a mixed-signal CNN that implements its first layer in the analog domain. Computing part of the operations of the algorithm using analog circuits degrades the accuracy by 1.3%, but it also reduces the power consumption by 15.7% because Analog-to-Digital Converters (ADCs) are one of the most power-consuming elements in standard CIS [57]. The SIS presented by Zhong et al. [29] computes edge detection and omnidirectional LBP using column-level circuits and array of capacitors capable of storing two rows of the image. Our own previous work [43] computes LBP using a combination on-pixel and column-level processing. An array of smart pixels performs the comparisons between adjacent pixels and outputs a binary value, which is used by column-level circuits to construct the LBP features. The single-bit output of the smart pixel allows us to reduce the memory required by the line buffers and the time to read the data from the pixel array and improves the fill factor by moving a significant part of the computation to the column-level circuit.

The discussion above shows that SISs are a viable alternative to digital processors to achieve the low power and high performance required by computer vision on mobile devices, while achieving comparable precision [37,43]. An SIS can exploit the pixel-level parallelism of the algorithm, but the area used by the processing circuits limits the fill factor of the SIS. This can be mitigated by performing part of the computation during photocurrent integration and by moving computation to column-level circuits. The design presented in this paper uses both techniques to build a two-mode imager that operates as a conventional sensor and computes object location, using a configurable CTIA suitable for the thermal IR range.

## 3. Object-Location Algorithm

**Figure 1:**
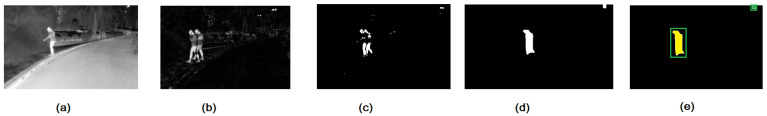
Results of the steps of motion-based object location: (**a**) input image, (**b**) result of the frame difference, (**c**) thresholding the frame difference, (**d**) applying the image-open morphological transformation, and (**e**) bounding boxes.

Our SIS performs object location using a multi-frame approach, which considers the information of consecutive video frames to locate moving objects [58,59]. Algorithm 1 summarizes the motion-based object location algorithm implemented by the SIS. It first computes the frame difference between pixels in consecutive video frames and compares this difference to a threshold to discriminate object pixels from the background. Then, the algorithm applies morphological operations to remove motion-detection artifacts. Finally, a connected-components algorithm computes the object bounding boxes from the motion pixel data. Figure 1 illustrates the operation of the algorithm, showing an input image and the output of each stage.


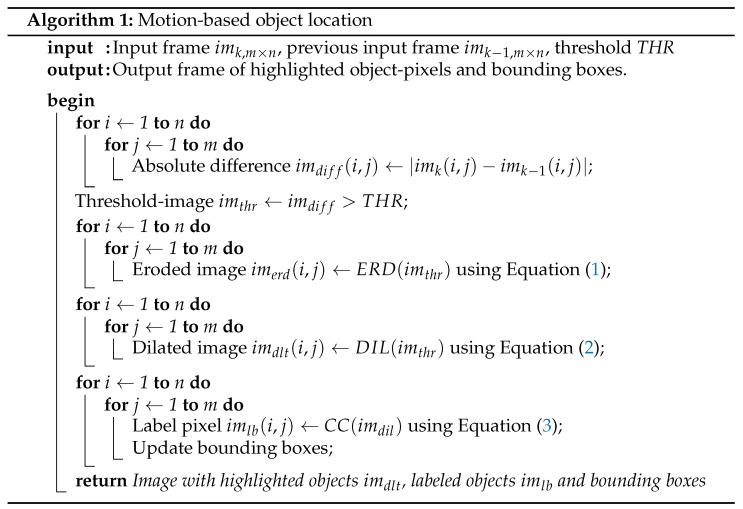


The frame-difference stage of the algorithm uses the same approach presented by Bir Bhanu et al. [60]. This algorithm detects pixels that belong to moving objects by comparing their value in consecutive frames. When the difference between the pixel value between two consecutive frames is higher than a predefined threshold, the algorithm assumes that the pixel belongs a moving object. This threshold is an application-dependent sensibility parameter that can be adjusted manually by the user. As shown by Yin et al. [61], an adequate threshold value can be determined by training the algorithm using a set of image data from the application environment.

The first stage of the algorithm computes the absolute frame-difference: it computes the absolute difference between corresponding pixels in two consecutive video frames. The next stage compares each pixel difference to an application-defined threshold: if the absolute frame difference is greater than the threshold, we identify it as a movement pixel and assign it a logic label of value 1. Otherwise, the pixel is labeled as 0. Figure 1c illustrates the output of the threshold stage. The figure shows that this method can produce isolated labels due to abrupt changes in pixel values. To compensate for this, it is common to add a morphological operation stage. We apply an image opening operation, which consists of image erosion followed by dilation. Figure 1d shows that this considerably reduces the number of isolated labels. Finally, Figure 1e shows, using different colors and bounding boxes, the output of the connected components algorithm, which labels the objects found in the image. From this point, it is possible to further extend image analysis to process shapes, single objects, and more.

To perform erosion and dilation, we used 3×3-pixel kernels. Since these morphological operations use single-bit pixels, the computation is reduced to simple logical AND and OR operations. Image erosion replaces the center pixel in a 3×3 window with the minimum value in the window. With binary images, erosion replaces the pixel with logical 0 if there is at least one pixel equal to 0 in the window (AND), as described in Equation (1):

(1) $$im_{erd}=ERD\left(im_{in}\right)=\left\{\begin{array}{cc} 0 & \mathrm{if}\;\mathrm{at}\;\mathrm{least}\;\mathrm{one}\;\mathrm{pixel}\;\mathrm{is}\;0\\ 1 & \mathrm{if}\;\mathrm{all}\;\mathrm{pixels}\;\mathrm{in}\;\mathrm{kernel}\;\mathrm{are}\;1 \end{array}\right.$$

Image dilation replaces the center pixel in a 3×3 window with the maximum value in the window. With binary images, dilation replaces the pixel with logical 1 if there is at least one 1 pixel in the window (OR), as described in Equation (2):

(2) $$im_{dil}=DIL\left(im_{in}\right)=\left\{\begin{array}{cc} 0 & \mathrm{if}\;\mathrm{all}\;\mathrm{pixels}\;\mathrm{in}\;\mathrm{kernel}\;\mathrm{are}\;0\\ 1 & \mathrm{if}\;\mathrm{at}\;\mathrm{least}\;\mathrm{one}\;\mathrm{pixel}\;\mathrm{is}\;1 \end{array}\right.$$

**Figure 2:**
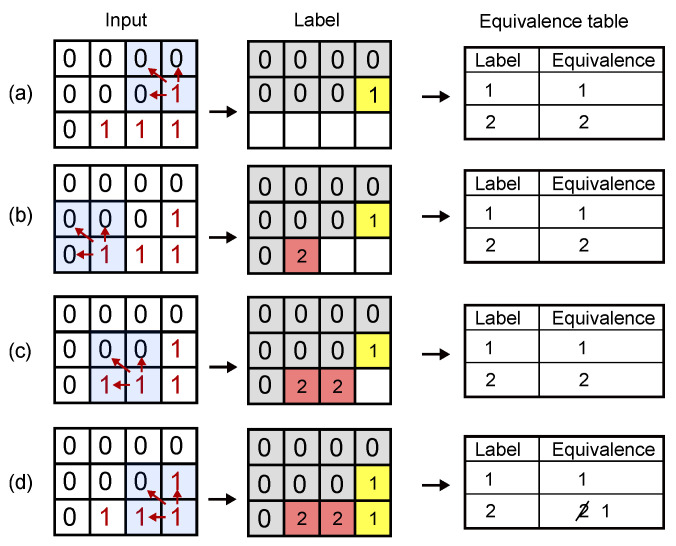
Graphic example of the connected components algorithm.

The third stage of the algorithm computes the bounding boxes for the objects located in the image in a single pass using a raster-scan connected components algorithm [62]. Figure 2 illustrates the operation of the algorithm. For each movement pixel in the image, the algorithm looks at its north, northwest, and west neighbors. If none of them are also a movement pixel, the algorithm assigns a new object label to the pixel, as shown in Figure 2a,b. Otherwise, if the neighboring movement pixels are part of the same connected component, the algorithm assigns the same object label to the new pixel, thus adding it to the connected component (Figure 2c). If the neighbor movement pixels belongs to different connected components, the algorithm assigns one of the labels to the new pixel and merges the connected components by adding a new entry into the equivalence table (Figure 2d). The base procedure of the algorithm is described as the priority-OR operation in Equation (3):

(3) $$im_{lb}=CC\left(im_{in}\right)=\left\{\begin{array}{cc} L_{0} & \mathrm{if}\;p_{s}\;\mathrm{is}\;0,\;\mathrm{or}\\ L_{i+1} & \mathrm{if}\;\mathrm{only}\;\mathrm{central}\;\mathrm{pixel}\;\mathrm{is}\;1\\ L_{nw} & \mathrm{if}\;\mathrm{central}\;\mathrm{and}\;\mathrm{north}-\mathrm{west}\;\mathrm{pixels}\;\mathrm{are}\;1\\ L_{n} & \mathrm{if}\;\mathrm{central}\;\mathrm{and}\;\mathrm{north}\;\mathrm{pixels}\;\mathrm{are}\;1\\ L_{w} & \mathrm{if}\;\mathrm{central}\;\mathrm{and}\;\mathrm{west}\;\mathrm{pixels}\;\mathrm{are}\;1 \end{array}\right.$$

where $L_{0}$ is the label for no-object pixels, and $L_{w}$, $L_{nw}$, and $L_{n}$ are labels of the west, northwest, and north pixels, respectively.

Every time the algorithm creates a new connected component or adds a pixel to an existing component, it updates the coordinates of its bounding box in a table. When the algorithm merges two connected components, it updates the bounding box.

## 4. SIS Architecture

**Figure 3:**
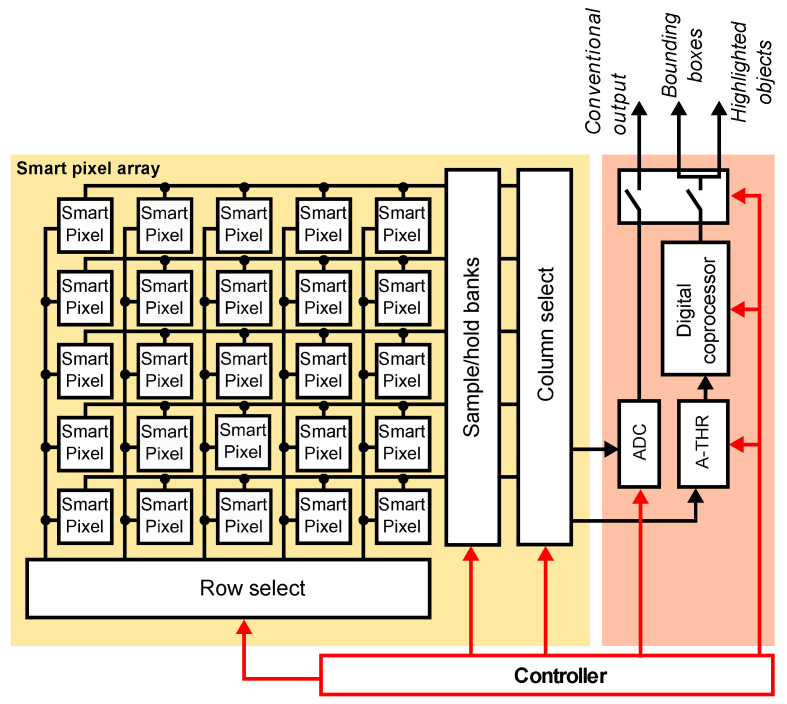
Architecture of the proposed SIS. An array of smart pixels outputs either the pixel value or the frame difference. The A-THR module determines whether the absolute value of the frame differences exceeds an application-defined threshold. The digital coprocessor computes image opening to improve the object location and uses a connected components algorithm to detect objects in the image and compute their bounding boxes. The digital coprocessor can be configured to output the original image or the binary image and the bounding boxes for the objects.

Figure 3 shows the architecture our proposed SIS. The SIS supports two operation modes: standard imager and motion-based object location. The core blocks are an array of smart pixels with local computational resources, an analog comparator (A-THR), and a digital coprocessor. In the standard mode, the pixel array acquires image data as a conventional image sensor, row- and column-select circuits sequentially read the pixel data, and an ADC produces digital values as a pixel stream. In object location mode, the SIS configures smart pixels to compute the frame difference, uses the A-THR to evaluate motion on each pixel and uses the digital coprocessor to determine the objects in the scene.

**Figure 4:**
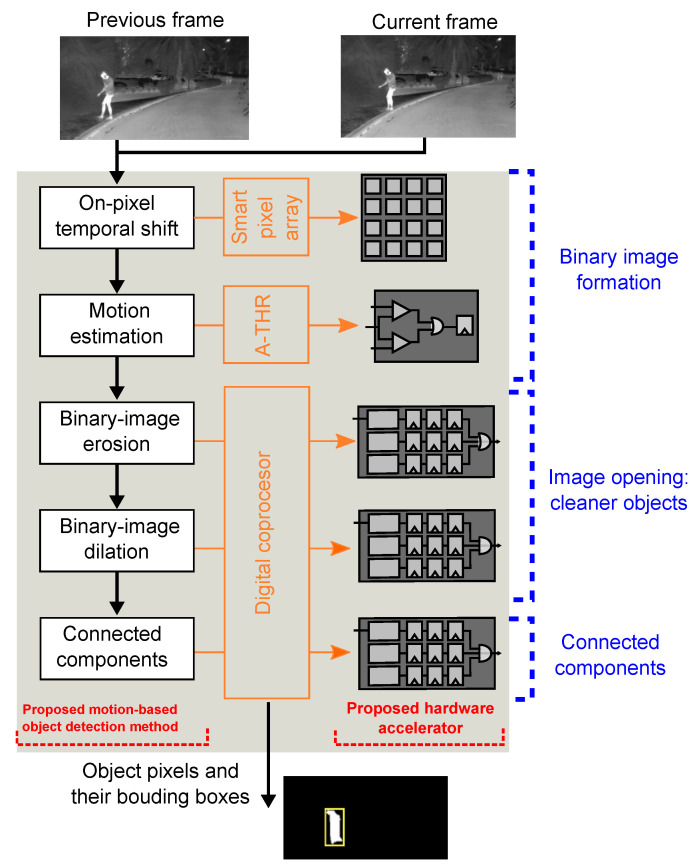
Illustration of the motion-based object location algorithm and proposed hardware accelerator.

Figure 4 shows a block diagram of our object location algorithm and the hardware modules of the proposed SIS that perform each step of the algorithm. In the center, the figure shows the three core blocks of the SIS, which are the smart pixel array, an analog comparator (A-THR) core, and the digital coprocessor. The first step of the algorithm is the temporal shift calculation implemented in the smart-pixel array. The second step is motion estimation, implemented on a custom analog-threshold circuit. The following steps correspond to the binary-image erosion and dilation and the connected components implemented on a digital coprocessor. With this configuration, the SIS computes the frame difference simultaneously along the array. In object location mode, the array acquires and stores the image data for the current frame and computes the difference between the current frame and the stored data for the previous frame. Then, the row- and column-select circuits sequentially read the frame differences and send them to the A-THR, which determines if the absolute difference of each pixel in the image is greater than the application-defined threshold. The output of the A-THR is a single bit for each pixel that serves as input to the digital coprocessor. The coprocessor computes the binary erosion and dilation, the connected components, and outputs the bounding boxes and binary image.

### 4.1. Smart Pixel

**Figure 5:**
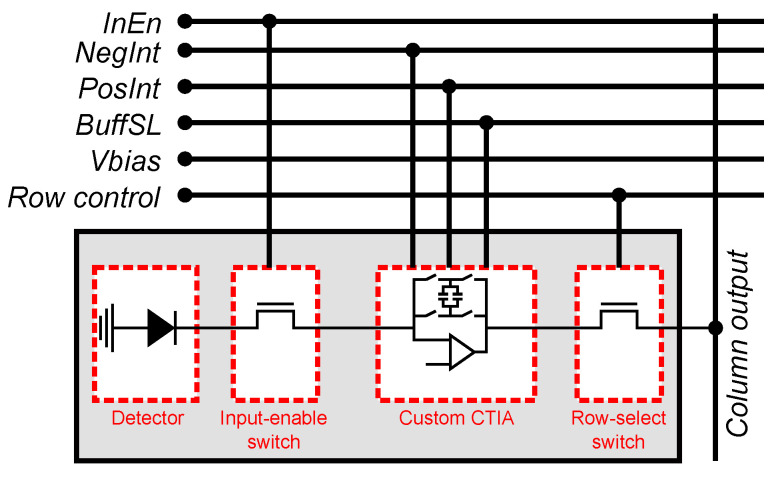
Architecture of the smart pixel. *Negtin*, *PostInt*, *BuffSL*, and *Vbias* are global bias and control signals. *Row control* is shared by all the pixels in a row and *Column output* is shared by all the pixels in the same column.

Figure 5 shows a block diagram of our smart pixel composed of a photodetector, switches for input enable and row-select, and a programmable CTIA. The input of the CTIA is connected to a photodetector. The CTIA uses the signals *NegInt*, *PosInt*, and *BuffSL* to integrate the photodetector current. The resulting voltage represents the value of the pixel in a frame or the difference between the pixel values in the current and past frames.

**Figure 6:**
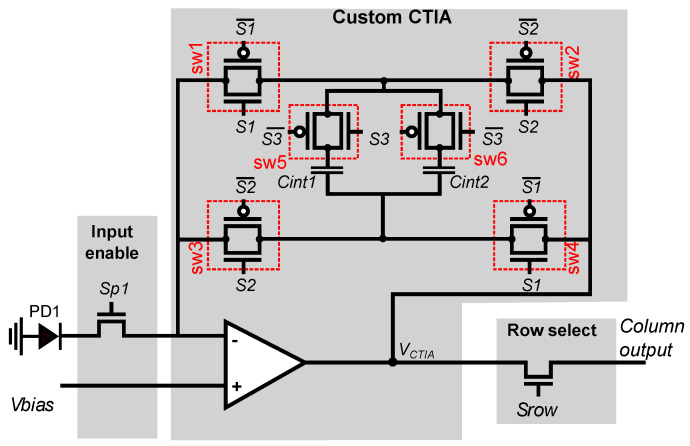
Configurable CTIA. The output voltage of the CTIA represents either the pixel value or the difference between the pixels in the current and past frame. Our configurable CTIA includes two integration capacitors of equal size, $C_{int1}$ and $C_{int2}$, which are used as double buffers to integrate and compute the frame difference.

A schematic view of the smart pixel is shown in Figure 6. The smart pixel is based on a conventional CTIA used for photodetector current integration, where we replace the single integration capacitor with two identical capacitors that act as a double buffer and six CMOS switches that select the active buffer and control the integration direction. The switches are controlled by three configuration signals that allow our custom CTIA to compute the difference between the pixel values in two consecutive frames during integration.

As described in [63,64,65,66], the CTIA is a preferred approach in two scenarios: environments with low light and IR cameras. Among the various types of circuits available in the literature, the CTIA configuration uses more area than many others for readout. However, as we described in our previous work [43], the CTIA has the following advantages: (1) low input impedance for good injection efficiency with weak photodiode currents, (2) less frame-to-frame latency, (3) wider linear output voltage range [67], (4) and reduced noise through better control of the photodiode bias [68].

**Figure 7:**
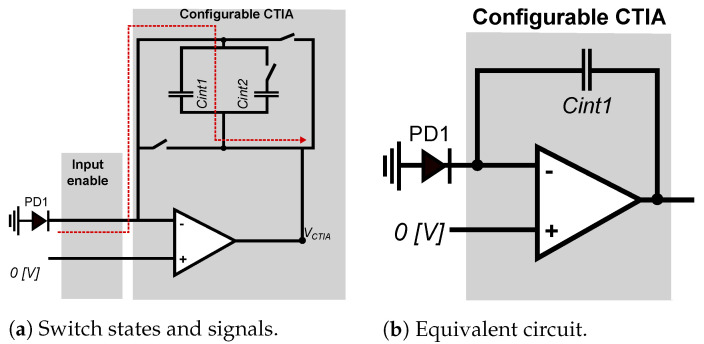
Smart pixel in conventional mode: the input-enable switch passes the current from the photodiode PD1, *sw1*, *sw4*, and *sw5* are closed to integrate the current using $C_{int1}$, and *sw2*, *sw3*, and *sw6* stay open.

The operation in conventional mode of our smart pixel is shown in Figure 7a, also referred to as direct mode. In conventional modem the operation of the smart pixel is equivalent to the conventional CTIA. For this operation, the smart pixel sets the bias voltage to 0V, and the CTIA integrates the input current on the capacitor $C_{int1}$. As shown in Figure 7b, in direct mode the smart pixel works as a conventional CTIA, where it sets the switches *sw1*, *sw4*, and *sw5* as closed and *sw2*, *sw3*, and *sw6* as opened. Equation (4) describes the output value at the end of the integration time:

(4) $$V=I\Delta t/C_{int1},$$

where *V* is the voltage at the output of the smart pixel, *I* is current from photodetector PD1, Δt is the amount of time that takes to integrate, and $C_{int1}$ is the capacitor value.

**Figure 8:**
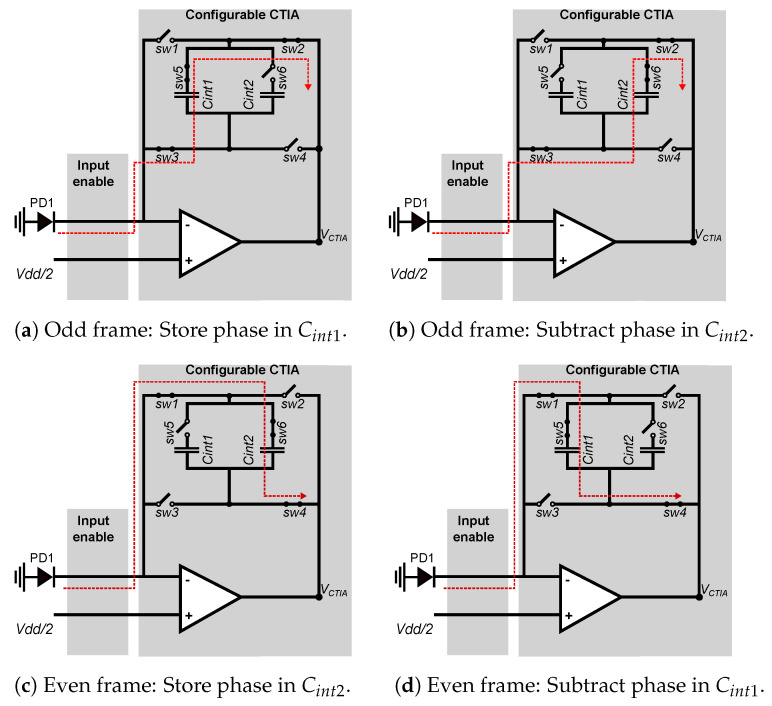
Simplified view of the CTIA in frame-difference mode during odd and even frames. During an odd frame, *sw2* and *sw3* are closed while *sw1* and *sw4* are open. During the store phase, *sw5* is open and *sw6* is closed, and during the subtract phase, the states off *sw5* and *sw6* are reversed. At the end of the frame, the voltage across $C_{int1}$ represents the frame-difference between the current and the previous frame. During even frames, the state of all switches is the complement of the odd frames, and the frame-difference is represented by the voltage across $C_{int2}$.

The operation of the smart pixel when computing the frame difference in the pixel is shown in Figure 8. The smart pixel sets the global bias input at the midpoint of the operation voltage. During a single video frame, the circuit operates in two stages: store and subtract, assigning one half of the integration time to each. During the store stage, the circuit integrates the input current into one of the capacitors, which will be used in the next video frame. During the subtract phase, the CTIA subtracts the input current from the second capacitor, which stores the pixel value of the previous frame. These stages operate in a slightly different way during odd and even frames because capacitors $C_{int1}$ and $C_{int2}$ operate as a double buffer: $C_{int1}$ is used to store pixel data (store phase) during an odd frame and to subtract the current pixel value (subtract phase) during an even frame. Conversely, $C_{int2}$ stores pixel data during an even frame and subtract the current pixel value during an odd frame.

Figure 8a,b show the equivalent circuits during an odd video frame. Here, the store and subtract phases integrate the input current in the positive direction in both capacitors: *sw2* and *sw3* are closed, and *sw1* and *sw4* are open. During the store phase, shown in Figure 8a, *sw5* is closed and *sw6* is open to integrate the input current on $C_{int1}$. During the subtract phase, shown in Figure 8b, *sw6* is open and *sw5* is closed to integrate (subtract) the input current on $C_{int2}$, which contains the pixel value acquired in the previous frame value. At the end of the frame time, the voltage across $C_{int1}$ represents the current pixel value to be used in the next frame, and the voltage across $C_{int2}$ is the frame-difference between the current and previous frames.

Figure 8c,d shows the equivalent circuits during an even video frame, which integrate the input current in the negative direction of both capacitors. The store phase integrates on $C_{int2}$ and the subtraction phase uses $C_{int1}$. The state of switches *sw1*–*sw6* is the complement of the odd frames.

At the end of the integration time, the CTIA outputs the voltage across capacitor $C_{int2}$ for odd frames and $C_{int1}$ for even frames, which represents the frame-difference value. The voltage across the capacitors at the end of the integration time is shown in Equation (5):

(5) $$V_{c}=\left(I_{k}\Delta t_{s}-I_{k-1}\Delta t_{s}\right)/C_{int},$$

where $V_{c}$ is the output voltage, *k* is the current frame index, $I_{k}$ is the input current during frame *k*, $I_{k-1}$ is the current during the previous frame k−1, $\Delta t_{s}$ is the integration time where $\Delta t_{s}=\frac{\Delta t}{2}$, and $C_{int}$ is the capacitance of $C_{int1}$ and $C_{int2}$. After the frame-difference values are read by the A-THR core, the circuit resets the capacitor that holds the frame-difference value.

Note that because we store and subtract in different directions during odd and even frames, the frame-difference in $V_{c}$ has a different sign in consecutive frames. This does not affects the results of the algorithm because the next stage uses the absolute value of the difference. Alternating the sign of the frame differences allows us to configure the CTIA using only three control signals. Moreover, sw1–sw6 switch only once per frame instead of once per phase, which reduces charge injection and power consumption.

Because the operation of the smart pixel divides the integration time into two stages of equal duration (store and subtract), it effectively reduces the integration time to 50% of the conventional mode. This decreases the signal-to-noise ratio of the imager but allows it to compute the frame-differences of all the pixels in the image in parallel.

### 4.2. A-THR

**Figure 9:**
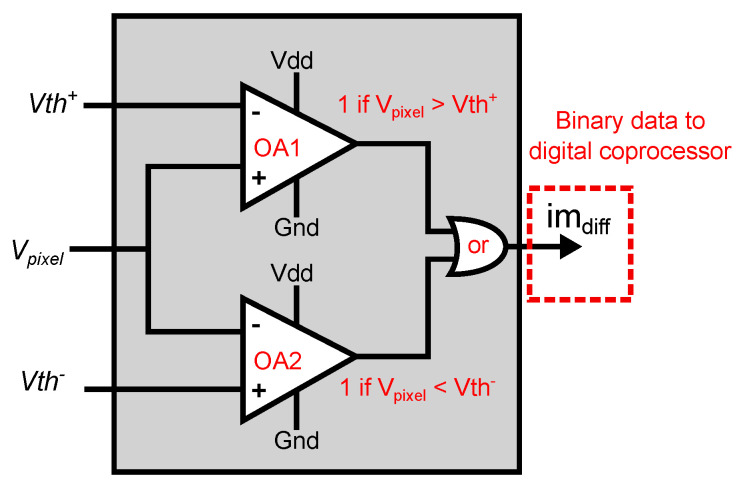
Architecture of the A-THR. An input comparator compares the frame difference for each pixel to two reference voltages. The comparator OA1 outputs a logical 1 when $V_{pixel}>V_{th}^{+}$, and the comparator OA2 outputs a logical 1 when $V_{pixel}<V_{th}^{-}$. A logical OR outputs a logical 1 if one of the two conditions is met.

The A-THR core determines whether the absolute value of the frame difference computed by the smart pixel exceeds an application-defined threshold. Figure 9 shows the A-THR circuit. A row- and column-select circuit scans the smart pixel array, reading the output voltage of each CTIA connecting it to the input $V_{pixel}$ of the A-THR module. Because the frame-difference output of the smart pixel has a different sign for even and odd frames, the A-THR core uses two comparators, OA1 and OA2. The reference voltages $V_{THR}^{+}$ and $V_{THR}^{-}$ are used to compare the absolute vale of the frame-difference voltage to the threshold voltage $V_{th}$, such that $V_{THR}^{+}=V_{bias}+V_{th}$ and $V_{THR}^{-}=V_{bias}-V_{th}$, where $V_{bias}=V_{dd}/2$ is the bias voltage of the CTIA in frame-difference mode.

The comparator OA1 outputs a logical 1 when $V_{pixel}>V_{th}^{+}$ and 0 otherwise, while OA2 outputs a logical 1 when $V_{pixel}<V_{th}^{-}$ and 0 otherwise. These two comparators independently indicate when $V_{pixel}$ is greater than $V_{th}^{+}$ or less than $V_{pixel}$. An OR gate outputs a logical 1 when either OA1 or OA2 outputs a 1, thus indicating that the frame-difference is greater than the supplied threshold. These logic values are generated for all the columns of the array in parallel and stored in a shift register. While the A-THR blocks process the next row, the shift register serially outputs the values from the previous row to the digital coprocessor.

### 4.3. Digital Coprocessor

The coprocessor adds programmability to the SIS by processing the output in frame-difference mode using reconfigurable digital logic. In our current implementation, the coprocessor implements the morphological opening operation and a connected components algorithm that detects objects and computes their bounding boxes.

**Figure 10:**
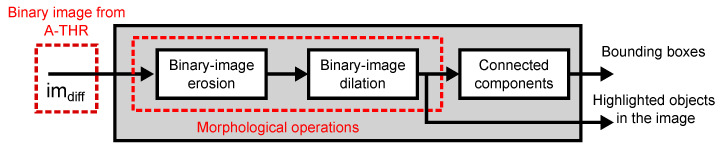
Architecture of the digital coprocessor. The coprocessor receives a stream of movement pixels, applies morphological opening operation (erosion+dilation), and computes the connected components of the resulting binary image and their bounding boxes.

Figure 10 shows the architecture of the object location coprocessor. The data flow of the digital coprocessor is as follows: the object location coprocessor receives a 1-bit pixel stream from the A-THR module. Then, the coprocessor computes morphological erosion and dilation operations in a 3×3-pixel window and outputs the resulting binary image. The image pixels are also processed by a connected components module, which identifies the objects in the image using connected pixels in a single pass and computes the bounding boxes of the objects.

**Figure 11:**
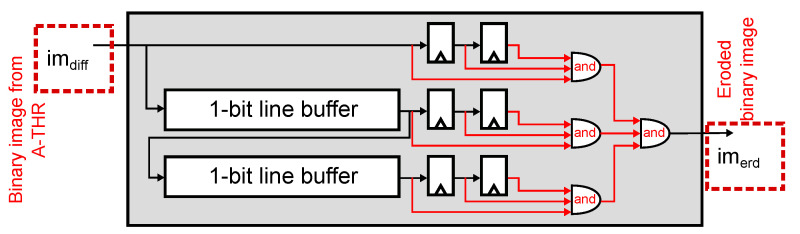
Image erosion. The module uses two line buffers and six registers to define a 3×3-pixel window from the output of the smart pixel array, and performs image erosion by computing a logical AND operation between them.

**Figure 12:**
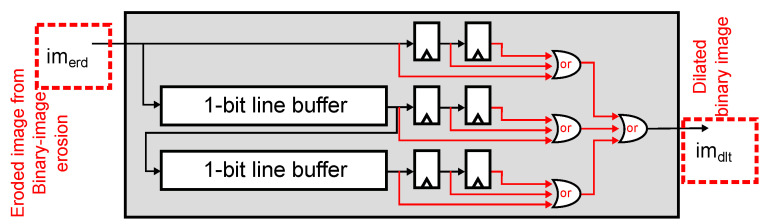
Image dilation. The module uses two line buffers and six registers to define a 3×3-pixel window from the output of the image erosion module and performs image dilation by computing a logical OR operation between the pixels.

The digital implementation of the 1-bit image erosion, defined in Equation (1), is shown in Figure 11. We erode with a 3×3 window by calculating the logical AND between all pixels in the window. We implemented the sliding window using two line buffers and a 2×3 array of Flip-Flops (FFs). Figure 12 shows the implementation of the 1-bit image dilation, defined in Equation (2). Dilation’s methodology is similar to erosion, which uses the same architecture but replaces the logical operations with OR gates.

**Figure 13:**
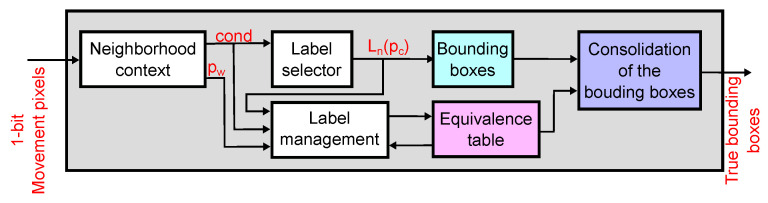
Architecture of the connected components module. First, it analyzes the current pixel and its north, northwest, and west neighbors, determining which movement pixels are connected. The module assigns a label to the current pixel and maintains an equivalence table to merge connected components in the image. The module also computes the bounding boxes for all connected components and merges them using the equivalence table.

Figure 13 shows the architecture of the connected components module. The Neighborhood Context block uses a line buffer to define a 2×2-pixel window that contains the current pixel and its north, northwest, and west neighbors. The block output indicates whether the current pixel is an isolated movement pixel, to which of its neighbors it is connected, or whether it is not a movement pixel. The Label Selector block assigns a new or existing label to the current pixel based on its neighboring labels, using a line buffer with label information. Because new pixels can join disconnected regions, the module uses an equivalence table to merge connected components. The Label Management block updates the equivalence table using the information from the neighborhood context. As new pixels are added to the existing connected components, the coordinates of their bounding boxes are updated using the contents of the equivalence table to consolidate regions as they merge.

## 5. Results

### 5.1. Smart Pixel and A-THR Implementation

To implement the smart pixel shown in Figure 6, we used minimum-size transistors to implement switches sw0–sw6. Switch sw0 uses an NMOS transistor, and switches sw1–sw6 are full transmission gates. The OPAMP in the custom CTIA and the comparators in the A-THR (Figure 9) use the design that we presented in [69]. The OR gate is a standard CMOS logic circuit.

**Figure 14:**
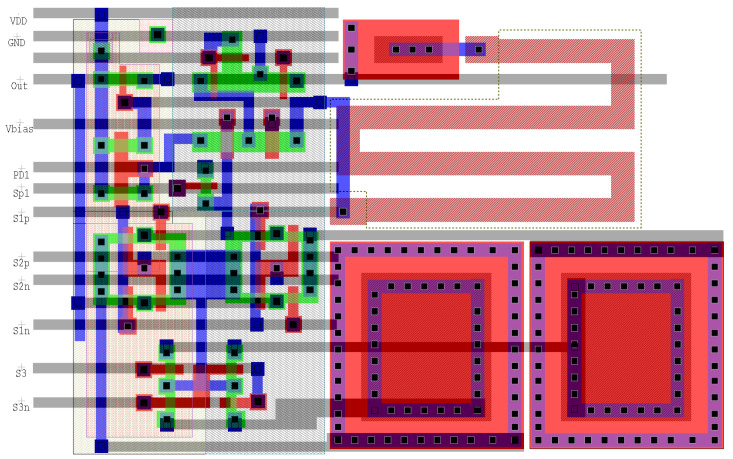
Diagram of the smart-pixel layout. We used the design shown in Figure 6, implemented on the TMSC 0.35 µm mixed-signal process. The Opamp and integration capacitors are implemented using two poly layers.

Figure 14 shows the physical layout for the smart-pixel as described above. This design uses a 0.35 µm mixed-signal process, 950 aF/µm${}^{2}$ poly1-poly2 capacitors, and a supply voltage of 3.3 V. The post-layout simulations with parasitic extraction presented in Section 5.2 were obtained using this process, for which we have access to the necessary technology files. The integration time of our smart pixel is 20 µs, and the maximum current that the photodetector delivers is 8 nA. With this, the two integration capacitors have an equal capacitance of 50 fF with a size of 7.7 µm × 7.7 µm. The area of the entire smart-pixel circuit of is 32 µm × 23 µm, which achieves a fill factor of 28% in a standard 32 µm × 32 µm pixel [40]. In comparison, a conventional CTIA circuit designed on the same process has a fill factor of 47.6%.

In order to assess the impact of technology scaling on the fill factor of the smart pixel, we redesigned the pixel using a 0.18 µm TMSC process, a technology commonly used in the literature [29,31,40]. For this technology, we used a supply voltage of 1.8 V and metal capacitors of 2 fF/μm${}^{2}$ capacitance. The size of the circuit is 14 µm × 19 µm, which achieves a fill factor of 74% in the same 32 µm × 32 µm pixel, compared to 86.3% with the conventional CTIA.

### 5.2. Simulation Results

**Figure 15:**
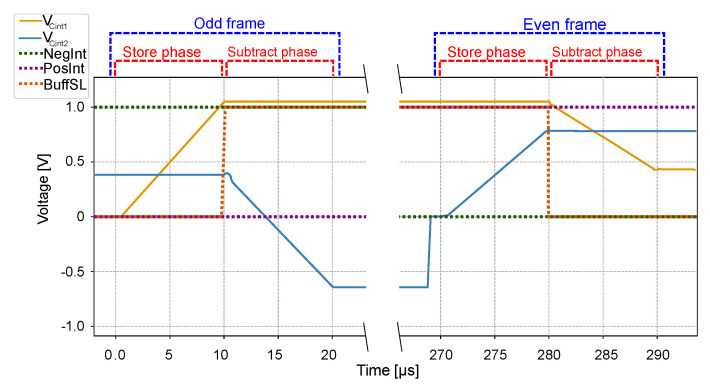
Post-layout simulation of a pixel in the SIS operating in frame-difference mode. The graph shows the voltage across the two integration capacitors of the CTIA during two consecutive frames.

To validate our smart pixel circuit, we simulated and tested it using a post-layout simulation of the circuit in Figure 14 using the 0.35 µm mixed-signal process. The simulation plot shown in Figure 15 depicts the main control signals *NegInt*, *PosInt*, and *BuffSL* (shown in Figure 5) and the voltage across the capacitors Cint1 and Cint2 during two consecutive video frames, while the SIS is operating in frame-difference mode. During the odd frame, *NegInt* and *PosInt* are set to 1 and 0, respectively, to configure the CTIA to operate as in Figure 8a,b. During the even frame, the circuit operates as in Figure 8c,d by setting *NegInt* and *PosInt* to 0 and 1. Within a frame, *BuffSL* switches the operation of the CTIA between the store and subtract mode. During the store phase of an odd frame, the capacitor voltage $V_{Cint1}$ stars at zero and increases linearly with the photodetector current, while the voltage $V_{Cint2}$ stores the pixel value of the previous frame. During the subtract phase, $V_{Cint1}$ stays constant, and $V_{Cint2}$ decreases linearly with the photodetector current. At the end of the phase, the output voltage of the CTIA is $V_{CTIA}=V_{Cint2}+V_{bias}$, which represents the difference between the pixel value in the odd frame and the previous even frame. During the next even frame, the role of the capacitors is reversed, and the circuit output is $V_{CTIA}=V_{Cint1}+V_{bias}$, which represents the difference between the pixel in the current frame and the previous odd frame.

Figure 15 shows that when *BuffSL* switches the capacitors $C_{int1}$ and $C_{int2}$ in the CTIA, the capacitor voltages show the effects of charge injection. This effect can be compensated in the A-THR block at each column by adjusting the threshold voltages $V_{THR}^{+}$ and $V_{THR}^{-}$.

**Figure 16:**
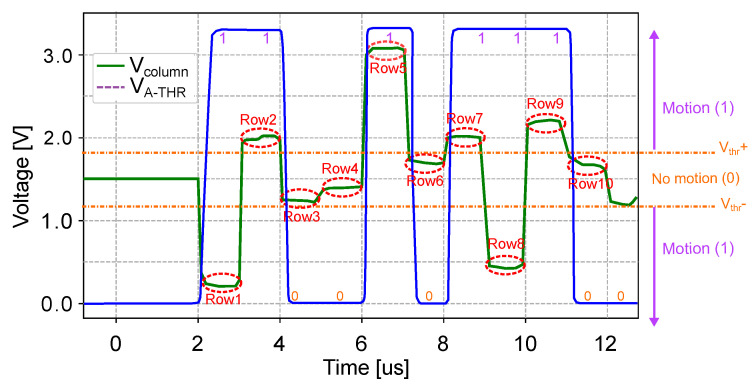
Post-layout simulation of the A-THR block while reading multiple pixels in frame-difference mode. The plot shows the subtract phase for two pixels A and B, in the same column during an odd frame. In the readout phase, the comparator consecutively samples all pixels in each column, comparing their value to the application-defined thresholds, and outputs a logic 1 when the movement in the pixel exceeds the threshold. Pixel values are sampled every 50 ns.

The plot in Figure 16 shows a post-layout simulation of the A-THR comparator shown in Figure 9. As described above in Section 4.1, after computing the frame difference during the subtract phase, all pixels contain their respective frame-difference value. Figure 16 shows the input voltage of the A-THR in one column, and its outputs voltage while reading pixel values in 10 consecutive rows. During the readout and comparison phase, the controller sequentially reads the CTIA outputs of each row in the column. The column voltage is labeled $V_{\mathrm{column}}$ in Figure 9. In this experiment, we sampled each row for 1 µs and circled in red the value of the pixels in each row. If $V_{\mathrm{column}}$ is outside the threshold window, i.e., $V_{\mathrm{column}}>V_{\mathrm{THR}}^{+}$ or $V_{\mathrm{column}}<V_{\mathrm{THR}}^{-}$, the A-THR outputs a logic 1. Otherwise, it outputs a logic 0. The shift register that captures the outputs of the A-THRs in each column operates at 320 MHz. The maximum frame rate achieved by the smart pixel array is given by the time to compute the frame-difference in the array (20 µs), plus the time to read 240 rows in parallel 240 µs. Therefore, the array can achieve a maximum frame rate of 3846 fps. At this frame rate, the smart pixel has a power consumption of 8.15 µW.

### 5.3. FPGA Implementation of the Digital Coprocessor

We used the SystemVerilog Hardware Description Language (HDL) at the Register-Transfer Level (RTL) to implement and validate the architecture of the digital coprocessor using the Xilinx Vivado 2020.1 development platform. In order to showcase the reduction in digital hardware resources enabled by the SIS, we targeted the low-cost entry-tier Xilinx Artix-7 XC7A35T FPGA. We compare our results to an FPGA-based Fully Digital Implementation (FDI) of the algorithm that uses a conventional image sensor. The FDI operates on 8-bit gray-scale pixels. All implementations use 5-bit labels and a 32-entry equivalence table in the connected components module. We consider two tests scenarios with different input image resolutions: 320×240 and 640×480 pixels.

Table 1 shows the resource utilization of both implementations for both image resolutions. Our proposed coprocessor architecture using the SIS requires 5930 and 3929 Lookup Tables (LUTs) for the 640×480- and 320×240-pixel implementations, respectively. This represents 28.5% and 18.8% of the LUTs available on the XC7A35T FPGA. Our implementations also utilize 12% and 7.7% of the available FFs. No on-chip Memory Blocks (BRAMs) are required in our SIS-based architecture. When compared with the SIS approach, the FDI needs a frame buffer to compute the temporal differences between pixels in consecutive frames, which is implemented with BRAM to avoid using an external memory chip, which would limit the performance of the algorithm and increase the overall cost of the system. Indeed, the 320×240-pixel FDI requires only a small increase in the utilization of LUTs and FFs but uses 38% of the available BRAM. Moreover, the 640×480-pixel FDI requires more BRAM resources than those available on the FPGA, and thus could not be implemented on the selected device. The small hardware utilization of our SIS-based coprocessor leaves ample resources available, even on an entry-level device such as the XC7A35T FPGA. These resources could be used to implement the additional image-processing algorithm on the output produced by the SIS.

Table 2 shows the power consumption of the coprocessor estimated by Xilinx Vivado. Operating with the 20 MHz clock frequency imposed by the sampling rate of the SIS, the power consumption of our coprocessor is 27 mW and 34 mW for the 320×240- and 640×480-pixel inputs, respectively. The coprocessor can operate at up to 125 MHz, which enables it to operate at up to 1627 fps on 320×240-pixel images while consuming 58 mW and at up to 406 fps on 640×480-pixel images while consuming 61 mW. In comparison, the FDI with 320×240-pixel input consumes 39 mW at 20 MHz, and 97 mW at its maximum clock frequency of 104 MHz. Here, the power consumption of the frame buffer, implemented as on-chip memory, is nearly 50% of the total dynamic power. At this frequency, the FDI can operate at up to 1354 fps.

### 5.4. SIS Object Location Performance

To test the performance of the motion-based object-location algorithm on our SIS, we used the OSU Thermal Pedestrian [70] and the Terravic Motion IR [71] datasets. Both contain video sequences in the thermal IR range. Table 3 summarizes the image size in pixels, the number of video sequences, and the total number of images.

To evaluate the object location performance of the SIS on each dataset, we used a simulation of the complete SIS circuit with post-parasitic extraction and the FPGA-based coprocessor described in Section 5.1. We developed a software implementation of the algorithm using floating-point arithmetic and used it as a baseline to evaluate the performance of the algorithm on the SIS.

**Figure 17:**
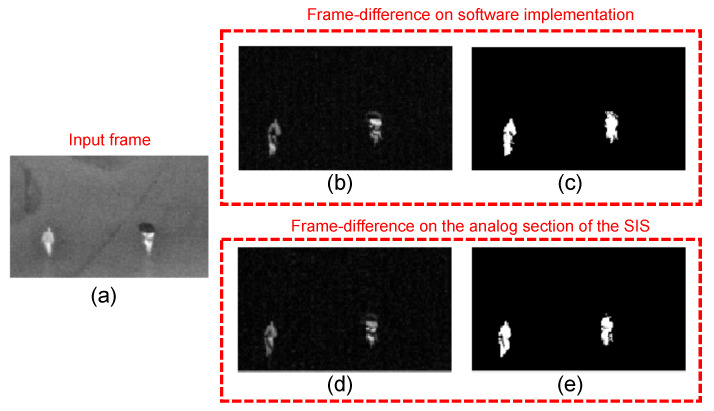
Visual comparison of the intermediate stages of the algorithm on the software and analog section of the SIS: (**a**) input frame, (**b**) frame-difference computed by the software, (**c**) software output after thresholding, (**d**) smart-pixel array output in frame-difference mode, and (**e**) A-THR output in the SIS.

Figure 17 shows a visual comparison of the intermediate stages of the algorithm on the software and the analog section of the SIS. Figure 17a shows the image input, taken from IR security footage in the OSU dataset, which shows two pedestrians crossing the street. Figure 17b,c show the absolute frame-difference and thresholding computed by the software, and Figure 17d,e show the same stages of the algorithm output by the smart pixel array and A-THR module in the SIS. The figure shows that both implementations produce visually similar results, although the SIS output loses resolution, mainly due to the reduction in integration time.

**Figure 18:**
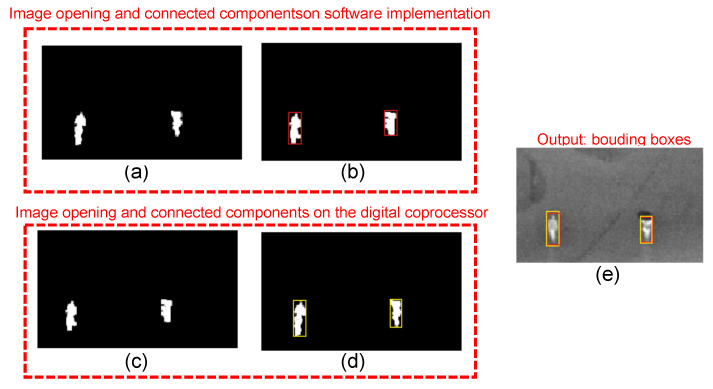
Visual comparison of the intermediate stages of the software and digital coprocessor: (**a**) image opening computed by the software, (**b**) bounding boxes in the software, (**c**) image opening computed by the digital coprocessor, (**d**) bounding boxes output by the digital coprocessor, and (**e**) comparison between the outputs of the two implementations.

Figure 18 shows a visual comparison of the intermediate stages of the algorithm on the software baseline implementation and the digital coprocessor. Because the two implementations receive a single-bit pixel image as input and the algorithm uses integer arithmetic only, they can produce identical results from the same images. However, the software and hardware implementations receive different inputs, as shown in Figure 17c,e. As a result, there are small differences in the image opening output (Figure 18a,c), which leads to differences in the bounding boxes (Figure 18b,d). Figure 18e overlaps the bounding boxes produced by the two implementations on the input image of Figure 17a.

We quantified the performance of the object location algorithm in the SIS implementation using the software implementation as a baseline. We used the Intersection over Union (IoU) index to estimate the accuracy of each bounding box output [72] and the average precision (AP), which measures the fraction of the objects in the image that are correctly located by the algorithm [72].

The IoU is defined in Equation (6) as:

(6) $$\mathrm{IoU}=\frac{area(SW\cap HW)}{area(SW\cup HW)},$$

where SW is the ground truth given by the bounding box computed by the software implementation, and HW is the same bounding box computed by our SIS hardware implementation. The IoU equals zero when the bounding boxes computed by two implementations have no overlap, and it equals one when the bounding boxes completely match. To compute the AP, we define a set of IoU threshold values $THR_{IoU}$, such that the location result of the $i^{th}$ object in the image is defined as a true positive (TP) when $IoU_{i}\geq THR_{IoU}$, and a false negative (FN) when $IoU_{i}<THR_{IoU}$. For each selected value of $THR_{IoU}$, the precision is computed as the ratio between the number of TP and the total number of objects (TP+FN) in the image. Finally, the AP of the algorithm is computed as the average between the precision values for each $THR_{IoU}$ in the image, for all images in the dataset.

Using the OSU dataset, we computed a total of 1050 bounding boxes from the 284 input images. The average value of the IoU for all boxes is 0.94. With the Terravic dataset, we obtained a total of 65,394 bounding boxes from the 23,355 images, for an average IoU value of 0.9. To compute the AP, we used $THR_{IoU}$ values in the range [0.85,0.95] with 0.01 increments. Our SIS implementation of the algorithm obtained an AP of 0.92 on the OSU dataset and 0.87 on the Terravic dataset.

### 5.5. Comparison to Related Work

Table 4 compares the smart pixel array proposed in this work to other designs reported in the literature, discussed in Section 2, that implement object detection on an SIS [30,40,56]. We also include our own previous SIS designed for face recognition [43], which also uses an iROIC to implement pixel-level operations.

The SIS presented in [40] detects objects using pixel-level processing to compute HOG features in an 8×8-pixel window. The processing circuits reduce the fill factor to 19%. The rest of the object detection algorithm is performed in a digital coprocessor and achieves an AP of 0.84. To improve the fill factor, the SISs in [30,56] move most or all the computation to the column level or to a coprocessor external to the imager. The SIS presented in [30] implements motion detection only to activate the digital coprocessor that performs object detection. The SIS combines pixel- and column-level processing to implement motion detection, and achieves a fill factor of 30% despite sharing capacitors between horizontally adjacent pixels. The coprocessor achieves an AP of 0.94. The SIS presented in [56] uses a digital coprocessor that operates at the column level, using an ADC for each column. Although it adds no additional circuitry at the pixel level, the die area used by the ADCs and coprocessors limits the fill factor to 60%. The digital coprocessor achieves an AP between 0.7 and 0.87, depending on the type of object detected.

Compared to works discussed above, our SIS achieves a frame rate that is significantly higher that those reported in the literature. This is mainly due to the parallelism exploited by our design at the pixel level and the fact that our column-level circuits have a single-bit digital output, which improves the readout time. Table 4 also shows that our fill factor is higher than those reported in the related work when using comparable CMOS processes. The main reason for this is that our SIS uses iROICs at the pixel level to compute the frame differences, which only add a capacitor and six extra switches to the conventional integration circuit. Finally, it is important to note that our design uses a CTIA to perform integration, which allow us to operate in the IR spectrum and low-light environments. The works reported in [30,40,56] only operate in the visible spectrum, but this allows them to use simpler pixel architectures with smaller die area.

The final column of Table 4 reports our own previous SIS [43] designed for face recognition, which uses an iROIC approach similar to this work. In consequence, the design achieves a similar fill factor, with slightly less area overhead because it uses only four switches and one capacitor per pixel. However, its maximum frame rate is significantly lower because it requires multiple reads per pixel to compute the features of the image at the column level.

Finally, we estimated a power consumption of 7.5 µW per pixel at 3846 fps for our design, which is higher than the power per pixel reported for other works in Table 4, although at a higher frame rate. The static power in the OPAMP of the CTIA is the main source of power dissipation and could be reduced by temporally powering down the CTIA when the array operates at a lower frame rate.

## 6. Conclusions

In this paper, we have presented the architecture and hardware implementation of an SIS for motion-based object location. The SIS uses a smart-pixel array with local memory to compute frame differences in the analog domain during pixel-current integration with high parallelism. It also uses an analog comparator and a digital coprocessor to compute image opening and connected components to detect objects from the frame-difference output of the smart-pixel array. We designed the smart pixel array and comparator at the layout level using the TSMC 0.35 µm and 0.18 µm mixed-signal CMOS processes and the digital coprocessor at the RTL level using SystemVerilog. We validated the design using post-layout simulations of the analog section and FPGA-based implementation of the coprocessor using a Xilinx XC7A35T FPGA.

Our results show that, using a 32 µm × 32 µm pixel, our design reduces the fill factor from 47.6% to 28% on the 0.35 µm process and from 86.3% to 74% on the 0.18 µm process, compared to a traditional imager. Because the integration time is reduced by 50% in frame-difference mode, the pixel resolution is decreased. However, the circuit can still detect objects with a mean IoU of 0.92 and an AP of 0.9 averaged over two thermal IR datasets, using a software implementation as a baseline.

Computing the frame differences on the smart-pixel array eliminates the need for a frame buffer in the digital coprocessor. Indeed, our results show that the FPGA coprocessor in our SIS does not use on-chip memory blocks, while a fully digital implementation of the algorithm requires 19 memory blocks for 320×240-pixel images and 75 blocks for a 640×480-pixel input. The latter cannot be implemented on the entry-level XC7A35T FPGA, which features only 50 memory blocks. The digital coprocessor attached to the SIS also achieves a higher maximum clock frequency, and therefore a higher frame rate, than the digital implementation of the algorithm.

When we use integration capacitors as double-buffer memory to compute frame differences, we reduce the penalty on the fill factor compared to circuits that operate with readout-circuit output. Furthermore, although our smart pixel effectively uses half of the integration time, which could reduce the signal-to-noise ratio, our results are comparable to a software implementation of the motion-based object location algorithm.

The on-imager computation of our SIS is convenient in contexts where privacy is required, where it eliminates the need to continuously transmit video information over a communication channel. Instead, the SIS can deliver an alarm only when objects in motion are detected. Another example is the use of our SIS paired with a high-resolution camera where the SIS could detect objects based on motion and send the bounding boxes to an external controller, which could use them to activate the capture of that portion of the high-resolution image.

## Figures and Tables

**Table 1:** Resource utilization of the digital coprocessor on a Xilinx Artix-7 XC7A35T FPGA.

	SIS 640 × 480		FDI 640 × 480		SIS 320 × 240		FDI 320 × 240	
	Used	%	Used	%	Used	%	Used	%
LUT	5930	28.5	6493	31.2	3929	18.8	4051	19.4
FF	5021	12.0	5107	12.2	3239	7.7	3270	7.8
BRAM	0	0	75	150	0	0	19	38

**Table 2:** Power consumption of the digital coprocessor on a Xilinx Artix-7 XC7A35T FPGA, estimated by Vivado. All implementations consume 20 mW of static power, which are added to the dynamic power to compute the total. The 640×480-pixel FDI can not be implemented on the XC7A35T device.

	Dynamic Power (mW)				Total Dynamic (mW)	Total (mW)
	Dilation	Erosion	Connected Components	Frame Buffer		
SIS 320 × 240 (20 MHz)	2	2	3	0	7	27
SIS 320 × 240 (125 MHz)	9	9	20	0	38	58
SIS 640 × 480 (20 MHz)	4	3	7	0	14	34
SIS 640 × 480 (125 MHz)	12	12	17	0	41	61
FDI 320 × 240 (20 MHz)	2	2	3	12	19	39
FDI 320 × 240 (104 MHz)	12	14	17	34	77	97

**Table 3:** Datasets used to test the performance of the proposed algorithm.

Dataset	Spectrum	Image Size	Number of Sequences	Total Number of Images
OSU Thermal pedestrian dataset [70]	Thermal IR	360×240	10	284
Terravic Motion IR dataset [71]	Thermal IR	320×240	18	23,355

**Table 4:** Comparison of our smart-pixel design to other circuits in the literature.

	This Work		[40]	[30]	[56]	[43]	
Technology (µm)	0.35	0.18	0.18	0.18	0.13	0.35	0.18
Array size (pixels)	320×240	320×240	256×256	256×256	320×240	150×80	150×80
Pixel pitch (μm)	32 × 32	32 × 32	31 × 31	5.9 × 5.9	5 × 5	32 × 32	32 × 32
Fill Factor (%)	28	74	19	30	60	34	76
Power (μw)	8.25 (pixel)	-	2.18 (array)	51.1 (array)	229 (array)	-	-
Type of integrator	CTIA	CTIA	OTA + 2 CAP	5T + 1 CAP	4T	CTIA	CTIA
Tested spectrum	IR	IR	Visible	Visible	Visible	Visible IR/NIR	Visible IR/NIR
AP	0.87–0.92	-	0.84	0.94	0.7–0.87	-	-
SIS fps	3846	-	30	30	15 (207 max)	556	-

## Data Availability

This study uses the following publicly available datasets: OSU Thermal Pedestrian Database http://vcipl-okstate.org/pbvs/bench/Data/01/download.html, and Terravic Motion Infrared Database http://vcipl-okstate.org/pbvs/bench/Data/05/download.html. All datasets were last accessed on May 2022.
